# How Learning Time Allocation Make Sense on Secondary School Students’ Academic Performance: A Chinese Evidence Based on PISA 2018

**DOI:** 10.3390/bs13030237

**Published:** 2023-03-08

**Authors:** Ang Liu, Yuguang Wei, Qi Xiu, Hao Yao, Jia Liu

**Affiliations:** 1 Faculty of Education, East China Normal University, Shanghai 200062, China; liuang@stu.ecnu.edu.cn; 2 School of Politics and Public Administration, Zhengzhou University, Zhengzhou 450001, China; wyg_swm@gs.zzu.edu.cn; 3 Institute of Higher Education, Tongji University, Shanghai 200092, China; 4 School of Economics and Management, Beihua University, Jilin 132013, China; liujia161716@163.com

**Keywords:** learning time, academic performance, threshold regression, PISA 2018

## Abstract

It is well known that the proper allocation of learning time is particularly important for promoting students’ academic performance. Based on the data from PISA 2018, this research used the method of threshold regression and quantile regression to explore the optimal length of learning time to promote the students’ academic performance. At the same time, this research also explored the heterogeneity of the effect of learning time on different academic levels of students. The results show that for four Chinese provinces and cities, including Beijing, Shanghai, Jiangsu province and Zhejiang province, students who study in rural areas and private schools usually have longer learning time than students in cities and public schools. Moreover, it is suggested that there is no significant association between school quality and students’ learning time. The average learning time of students from the four Chinese cities and provinces is obviously longer than that in OECD countries. Moreover, it is found that the impact of learning time on academic performance across subjects is inverted U-shaped, and the optimal study time can be found in the learning of mathematics, science, and reading related subjects. As for the effect of learning time, the results showed that learning time commitment is more effective for students who are academically disadvantaged. At the same time, this study found that there is a relationship between students’ excessive learning time and students’ subjective well-being and attitudes toward learning activities. The non-cognitive factors can influence students’ academic performance gradually. According to the results of this research, it is suggested that students need to balance their learning time allocation against the appropriate learning time standards. Moreover, schools should adopt different learning time allocation schemes for students at different academic achievement levels. The teachers also should uphold a more scientific design of students’ after-school homework, and teachers and parents should also focus on improving students’ learning efficiency.

## 1. Introduction

Academic achievement has been regarded as an important issue in educational research. The importance of academic achievement to individual development is undoubted, especially in the increasingly competitive society [1]. In the elementary education stage, the students’ academic achievement is related to a variety of factors, such as students’ individual intellectual talent, family background, school education quality, and individual learning status [2,3]. When discussing individual learning status, learning duration is regarded as one of the most important influencing factors. It is commonly believed that investing more learning time must bring better academic performance [4]. In traditional Chinese culture, many sayings such as “Practice makes perfect (Qin Neng Bu Zhuo)” and “There is no royal road to learning (Shushan Youlu Qin Wei Jing)” indicate that devoting more time to learning usually leads to better academic success. However, at the same time, excessive attention to students’ learning time has resulted in too many academic burdens for students, and the interventions of families and schools on students’ learning time have caused excessive “involution”, and “Education Anxiety” is prevalent among families and societies. In order to curb this prominent problem, in July 2021, the Chinese government implemented a strong policy named “Guidelines to Ease the Burden of Excessive Homework and Off-campus Tutoring for Students Undergoing Compulsory Education” (also called “Double Reduction Policy”) in the stage of compulsory education in order to create a better educational ecology through reducing students learning burden. With this background, it is essential to know how much time is currently invested in secondary school students’ daily learning in mainland China. Does the length of students’ learning time vary from region to region and from school to school? Is the learning time of Chinese secondary school students too much compared with that in OECD countries? What should be the ideal amount of learning time for secondary school students? Is the ideal allocation of study time different for students at different academic levels (for example, the difference between gifted students and underachievers)? In this research, the data from PISA 2018 are used to reflect on how to promote the reasonable allocation of learning time for Chinese secondary school students. This research also aims to provide an empirical basis for international education policy making.

## 2. Literature Review

In the 1970s, with the globalization development of compulsory education, scholars started to explore the relationship between learning time and academic achievement, and it is well realized that learning time is an important factor affecting students’ academic achievement indeed [5]. However, there are still some controversies and uncertainties about the relationship between students’ learning time and academic achievement.

Some of the research suggested that there is a positive correlation between students’ learning time and their academic performance and achievement. For example, through analyzing 15 major empirical studies on related topics in the United States over the past 24 years, Patall and colleagues found that longer course time was effective in improving students’ academic performance in all subjects, especially for the students who have a lower academic performance [6]. Moreover, research conducted by Masui and colleagues claimed that for most courses, longer learning time usually predicted a better academic performance even after accounting for the students’ personal characteristics (e.g., gender, reading ability, etc.) [7]. Furthermore, Mullis et al. claimed that there is a positive correlation between the length of mathematics instruction and students’ academic performance in mathematics by analyzing the result of the International Mathematics and Science Study Survey (TIMSS). The result also reflects that more course input compensated for students’ cognitive deficits [8]. The research by Ozyildirim also showed that students’ after-school homework completion time has an impact on academic performance [4], with an effect size of Cohen’s d = 0.186 between the two variables, indicating a small but significant positive relationship between after-school homework completion time and students’ academic performance. Gronder’s research also claimed that additional homework time would improve students’ academic achievement, especially for lower-performing students [9].

Additionally, some research results show that learning time and learning outcomes are negatively correlated. For example, according to Kember et al.’s research, the result shows that even though students’ learning time is an average of up to 65 h per week [10], they still receive lower grade point averages (GPAs) when they mindlessly and repetitively solve the questions without really understanding the course content in a meaningful way. Plant et al. found that the total learning time was negatively correlated with students’ GPA and scores on the SAT by recording the amount of time students spent studying on campus (at home) and off campus (in the library, etc.) [11].

Some studies suggest that there is a nonlinear relationship between learning time and academic performance. Tang and Fu proposed an inverted U-shaped relationship between homework time and their final subject grade [1]. The students from grade four and grade seven could achieve optimal performance by completing homework within 1 to 2 h per day, but their performance declined if the learning time was less or more than this time frame. The research conducted by Cooper also found a nonlinear relationship between homework time and academic performance, and it is demonstrated that there is a reasonable interval of homework volume [12]. When the homework time is in the optimal interval, students achieve the most desirable learning outcomes, and when the ideal homework time interval is exceeded, the impact of homework on academic performance will show a decreasing trend. The latest research on PISA found that there is an inverted U-shaped quadratic relationship between learning time and students’ science grades. The students’ science grade become improved with the increase in learning time investment and then decreased with the increase in the students’ learning time, and this fluctuation of achievement difference with time extension was more obvious in East Asian countries [13].

However, several studies have argued that the relationship between student learning time and learning outcomes is uncertain, with differential effects across individual characteristics. It is suggested that the effects of learning time are heterogeneous, especially for students with different learning abilities [7]. Moreover, by comparing the US local student and international students, Eliasson et al. found that although international students have 50 min more learning time per day than native students, their GPAs did not differ significantly [14]. Furthermore, the research conducted by Everaert suggested that the relationship between learning time and academic performance was not always directly correlated, and this kind of relationship could be influenced by students’ learning ability, specific subject characteristics, and environmental factors [15]. Kalenkoski’s research further suggested that the length of students’ learning time usually had different effects on the academic performance of students of different genders [16]. Boys generally spend less time on study than girls, and the increase in learning time can usually slightly increase boys’ academic performance but does not have any positive effect on girls’.

There are two different theoretical perspectives on the impact of learning time investment on students’ learning effectiveness; one is the Second Reward Theory proposed by Eisenberger, which argues that academic diligence is mainly expressed as the actual learning time investment [17]. The individuals will receive more feelings of diligence through more diligent behaviors. This kind of feeling produces secondary reward characteristics and encourages individuals to produce better academic performance [18]. Certainly, the prerequisite of this theoretical perspective is that individual students themselves have self-motivation to improve their academic performance to produce positive emotions rather than passively increase their learning time under external pressure. Another perspective is the relative learning time input theory, and the typical representative of this orientation is Carroll [19]. Carroll proposed a learning input theory mediated by learning time input, which takes learning time as the most important variable influencing learning input. It is believed that the more time students invest in learning, the better learning engagement level the students will have. The prerequisite of this theory is that the time invested in learning should be controlled within the necessary time frame. A negative effect on students’ academic performance will be produced if the learning time exceeds the reasonable frame. Based on the two theories, it is necessary to analyze whether there is an optimal range of learning time for secondary school students.

Current studies have found some effects of student learning time allocation on student academic performance; however, the shortcomings are still there. Firstly, there are no related research studies based on developing countries, especially China, where the education context has long been influenced by Confucianism. Chinese secondary school students have actually long been controlled by learning time commitment both at school and home with a highly competitive academic environment. Based on this background, the effect of learning time allocation on student achievement deserves to be explored. Secondly, the optimal length of learning time to promote students’ learning outcomes in each course has not been explicitly explored, and specific learning time standards need to be measured to provide an empirical basis as a reference to promote the students’ achievement. Moreover, there is very rare research focusing on the heterogeneity of learning time allocation for students with different levels of learning outcomes. Some studies found a positive or inverted U-shape correlation between learning time and academic achievement, but it is worthwhile to investigate whether the same effect exists on students with different learning abilities and whether differentiated learning time allocation strategies are adopted for students with different academic abilities. Therefore, this research makes precisely the above research contributions by analyzing the optimal learning time that promotes students’ learning outcomes through threshold regression and analyzing the heterogeneity of the effects of increased learning time on different students through using quantile regression.

## 3. Materials and Methods

### 3.1. Data Sources

The data of this research were obtained from the test results of The Program for International Student Assessment 2018 (PISA 2018), a worldwide ability assessment program conducted by OECD for 15-year-old students. The data of this research come from the OECD website (https://www.oecd.org/pisa/) (accessed on 8 November 2022) and contain data sources, technical solutions, and data descriptions. The PISA data collection is scientifically based on a rigorous two-stage sampling process. The first stage of sampling selected a representative sample of at least 150 schools in each country, taking into account regional disparities in educational development. The second stage of sampling randomly selected approximately 35 15-year-old students from the representative sample of schools to participate in the assessment, with most countries assessing a sample of between 4000 and 8000 students. Because of the potential for sampling and measurement error, the PISA assigned a certain sampling weight to each sampled school and student. To date, PISA has conducted seven large-scale and global assessments of educational quality, and the surveys are designed and implemented by international experts involved in the assessment; therefore, the validity and credibility of the data can be assured [20].

In PISA 2018, there are four cities and provinces, including Beijing, Shanghai, Jiangsu, and Zhejiang, which have taken part in the assessment, and the assessment items include the related issues of schools, students, and their families. Due to the sampling and measurement errors, each student and school in the PISA sample is assigned a certain sampling weight, which is also included in the OLS regression model. After excluding the corresponding missing or invalid samples, this research finally selected a sample of 9343 students from the PISA 2018 database.

### 3.2. Variables

According to previous experience, the research on student academic performance is generally analyzed based on input–output efficiency models, and Hanushek’s classical educational production function theory model has been commonly used. The factors influencing students’ achievement output (*Y_ij_*) can be separated into two main factors. The first factor is personal characteristics such as innate learning ability (*I_ij_*), family background (*F_i_*_j_), school education quality (*SC_j_*), and student learning time (*Time_ij_*). The second factor refers to the other random residual components that cannot be estimated (*e_ij_*). The theoretical model developed is as follows:

(1) $$Y_{ij}=F(I_{ij}+F_{ij}+SC_{ij}+Time_{ij}+e_{ij})$$

Based on the theoretical model, the independent variables, dependent variables, and control variables selected by this research are shown in Table 1. The descriptive statistics of the variables are also shown in Table 1.

#### 3.2.1. Dependent Variables

The dependent variable in this article is student academic performance, which is expressed as the grade of three subject tests in PISA: mathematics, language, and science. The Item Response Theory (IRT) model is used to estimate the probability distribution of each student’s performance in the subject tests after repeating 10 times to form 10 plausible values for each student in the three key competency tests. At the same time, the weights of each student’s grade on the subject competency test were assigned. In this research, the maximum likelihood estimation was adopted in the OLS model by using STATA software, incorporating 10 plausible value estimates for each student. At the same time, the student weights and school weights of PISA data were also used in this research.

#### 3.2.2. Independent Variable

The independent variable of this research is student learning time. It refers to the length of time students devote to learning each subject course with the unit of minutes/week. PISA data reported the students’ learning time in math, language, science, and also total learning time. It mainly refers to the learning time invested in a particular subject or in all of the subjects. It includes the total learning time both inside and outside the classroom. The learning time investment measured by the PISA also excludes the time wasted on non-instructional activities, so the learning time refers to the time students spend on real learning. In terms of variable correspondence, math learning time corresponds to the students’ math subject performance, language learning time corresponds to students’ reading subject achievement output because language learning is one of the necessary conditions to promote reading performance, and science learning time corresponds to science subject achievement output.

#### 3.2.3. Control Variables

Family background. It was demonstrated that family background factors such as family socioeconomic, cultural, and social status affects student achievements [21], and when analyzing the influence of students’ learning time on students’ academic performance, it is necessary to control the factor of student family background, which is measured by the family economic, social, and cultural status (ESCS) of the PISA data. It can characterize the overall level of a student’s family background.

School quality. Teacher quality is an important factor reflecting the quality of school education, and previous studies have claimed that teacher quality is a core element affecting students’ academic achievement [22]. The average level of teacher qualifications is the comparable and widely used indicator to distinguish inter-school quality differences. It is an important evaluation indicator of teachers’ human capital and an important input indicator to measure the quality of the school’s teaching force [23]. Therefore, this research selected the proportion of teachers with a master’s degree as the core proxy variable to indicate school quality. This indicator was sample aliquot cut to obtain high-quality and regular schools, where the average proportion of teachers with a master’s degree in high-quality schools was 24%, and the average proportion of teachers with a master’s degree in regular schools was 4%.

Innate cognitive ability. In addition to the influence of family background and the quality of teachers in schools, individual innate cognitive ability is also important in influencing students’ academic performance. Therefore, this research selected comprehension and memory metacognitive ability in PISA as proxy variables for inner cognitive ability.

Gender, school location, and school background variable. Individual gender, urban or rural district, and school type may all have an impact on students’ academic performance. The gender difference may affect individual preferences on different subjects [24]. There are also some arguments on this topic, and it is claimed that gender has a differential impact on knowledge, performance, and skills [25](Salanova et al., 2010). Moreover, differences in educational resources between urban and rural areas may lead to achievement differentiation [26]. Regarding school type, which includes public schools and private schools, the students’ performance of learning time and academic performance are also different due to the different management autonomy of different types of schools. Therefore, school type was also included as a control variable.

#### 3.2.4. Mediating Variables

Regarding the mechanism of the influence of learning time on academic performance, it has been shown that moderate learning time usually indicates a positive emotional experience, which is suggested will promote students’ learning efficacy and then make the students produce higher learning efficiency [27]. However, at the same time, it has been demonstrated that excessive learning time usually has a negative effect on students’ psycho-emotional feelings, such as their self-efficacy [28]. Additionally, some studies suggest that subjective attitudes and well-being have an influence on students’ academic performance [29,30]. Therefore, in exploring the mechanism of the effect of learning time on academic achievement, this research selected students’ psycho-emotional factors as mediating variables, and two variables, including attitude toward school (learning activities) and subjective well-being (sense of belonging to school), were measures as the mediating variables. This research hypothesizes that the effect of learning time on both of the two variables is in an inverted U-shaped relationship, which is positive within the moderate time but negative beyond a certain range, which in turn affects students’ academic performance.

### 3.3. Data Analysis 

There are four steps in data analysis. (1) The research explores the effect of students’ learning time on academic achievement through the OLS multiple regression model. (2) This research analyzed the optimal range of students’ learning time through a threshold regression model. (3) The Quantile regression model was used to analyze the heterogeneity of the learning time investment for students with different academic performance levels. (4) A two-stage OLS regression model was used to analyze the mechanism of the influencing mechanism of learning time on students’ academic performance. All of the data analyses were analyzed by STATA 16.

#### 3.3.1. OLS Multiple Regression Model

An OLS multiple regression model was selected to explore the effect of learning time on students’ academic performance. Since the dependent variable academic achievement is a continuous variable, an ordinary least squares regression model (OLS) was used to estimate it. The independent variables gender, urban and rural classification, and school type are all dichotomous variables, and they are treated as dummy variables of 0 and 1. For gender, female is regarded as the reference group; for urban–rural classification, rural area is regarded as the reference; and for school type, the private school is regarded as a reference group. All other variables are continuous variables and can be placed directly into the model. The estimated coefficients of the model indicate the value by which the dependent variable can increase/decrease for each unit increase in the independent variable. The basic econometric model for the study was set as follows:

(2) $$Y_{ij}=\beta_{0}+\beta_{1}I_{ij}+\beta_{2}F_{ij}+\beta_{3}SC_{ij}+\beta_{4}Time_{ij}+\varepsilon_{ij}$$

where *Y_ij_* is the output of student performance in each subject, *I_ij_* is the individual background characteristics such as gender and urban–rural classification, *F_ij_* is the family socioeconomic and cultural status index, *SC_j_* is the school quality, *Time_ij_* is the student learning time on each subject, and *e_ij_* is the residual.

#### 3.3.2. Threshold Regression Model 

Ordinary OLS regression models, as well as quantile regression models, only consider the linear effect of independent variables on students’ resilience ability. However, in this research, it assumed that there is an optimal range threshold for the learning time commitment. Moreover, the effect of learning time on student academic performance may not be presented singularly in a linear style; actually, it is in a dynamic process of change. In order to demonstrate whether there is a threshold effect on the change in students’ academic performance due to the change in learning time, this research constructs a threshold regression model to explore the nonlinear relationship between the effect of students’ learning time on students’ academic achievement. The “threshold” code is used on the threshold regression, and the threshold values are set to 1 and 2, respectively. The threshold effect is analyzed through the threshold test. The model equation for the threshold regression is as follows:

(3) $$Y_{i}=\alpha_{i}+\beta_{11}^{\prime }X_{1}D\left(qi\leq \varphi \right)+\beta_{12}^{\prime }X_{1i}D\left(qi>\varphi \right)+\beta_{2}^{\prime }X_{2i}+\varepsilon_{i}=\alpha_{i}+\theta X_{i}\left(\varphi \right)+\varepsilon_{i}$$

In this model, *Y_i_* refers to student resilience which is regarded as the dependent variable in this research. *X*_1*i*_ refers to the core explanatory variable influenced by the threshold, *X*_2*i*_ refers to the non-core explanatory variable not influenced by the threshold, *φ* is the real threshold value estimated by proxy, and *qi* and D(X) denote the threshold variable and the indicative function, respectively. In this research, the threshold variable *qi* is student learning time, and while either of the threshold variables is analyzed, the other variables are considered control variables. If the test finds that there is indeed a threshold for the learning time investment, it indicates that there is a nonlinear effect of learning time on students’ academic performance, and the optimal range of student learning time can be suggested based on the threshold value.

#### 3.3.3. Quantile Regression Model 

In order to estimate the heterogeneity of the effect of learning time on students at different academic ability levels, a quantile regression model was introduced for estimation, which has the advantage of accurately estimating the range of variation and conditional distribution characteristics of the independent variable on the dependent variable. Traditional regression analysis explores the relationship between the independent variable and the conditional expectation of the dependent variable; the correspondingly obtained regression model estimates the conditional expectation of the dependent variable; and quantile regression is usually used to analyze the relationship between the independent variable, the conditional quantile of the dependent variable, and the correspondingly obtained estimated conditional quantile of the dependent variable. For this research, the quantile regression model helps to reveal how student learning time affects student achievement levels at different quartiles and then to adopt precise and differentiated supportive strategies to improve the academic performance of students at different levels and to determine how student time investment should be allocated for students at different levels (gifted student and underachieved students). The code of “sqreg” is used in quantile regression. The quantile regression equation is expressed as follows:

(4) $$Q_{\theta }(y\left|X\right.)=Min\beta_{q}\sum_{i:yi\geq xi\beta q}^{n}q\left|yi-xi\beta q\right|+\sum_{}^{n}i:yi\leq xi\beta q\left(1-q\right)\left|yi-xi\beta q\right|$$

#### 3.3.4. Phased OLS Regression Model

**Figure 1:**
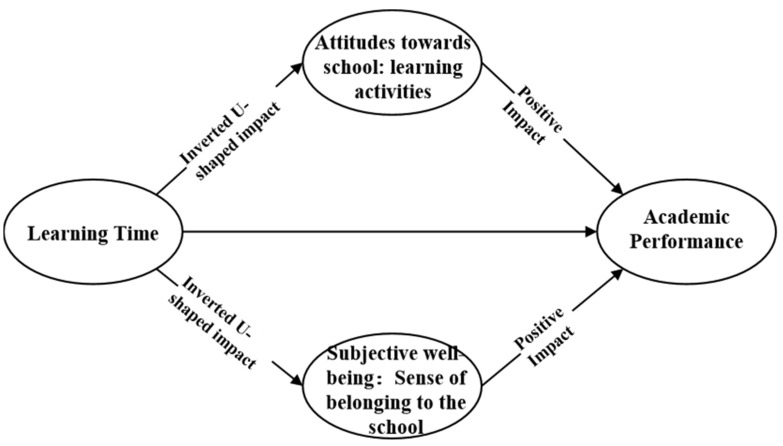
Analysis of the mechanism of learning time on students’ academic performance.

Based on the phased OLS regression model, the first stage incorporates the quadratic term of learning time, which is used to analyze whether the effect of study time on the two variables, including attitude toward school (learning activities) and subjective well-being (sense of belonging to school), and the second stage was used to analyze the effect of the two above variables on students’ academic performance, thus testing the hypothesized relationship in Figure 1. The code of “reg” is used in the Phased OLS regression model through STATA, and the OLS regression model runs in two stages.

## 4. Results

### 4.1. Learning Time Difference among Four Chinese Provinces and Cities

Through the data analysis, it can be found that there are differences in students learning time from different backgrounds, regions, and countries. As shown in Table 2, in terms of gender, there is no significant difference in the length of learning time between male and female students. As for the students from urban and rural areas, there is a significant difference in the length of learning time between students from these two areas, with students in rural areas having slightly longer learning time than their urban counterparts. The average learning time of rural students is 33 h per week, and while the learning time of urban students is 31.6 h, rural students have approximately 4% longer learning time. However, due to the more sufficient educational resources of the urban area and the higher efficiency of teaching, although the students in urban areas invest less time, they still perform better than rural students [31]. Regarding school type, students in private schools pay significantly longer time than students in public schools, with private school students having approximately 5% longer learning time. Public schools in China are regulated by the government, and adding extra learning time for students is not permitted. However, private schools have more autonomy, and some schools may mandate extra learning time for students during the night. In terms of teacher quality (differentiated by the proportion of teachers with a master’s and above degree in the school), the average length of learning is shorter for students in schools with higher-quality teachers, but there is no statistically significant difference. Regarding international comparisons, the average length of learning is significantly longer for students in China than that in OECD countries, with approximately 17% longer for students in Chinese schools. Chinese secondary school students have much longer learning time indeed than that in OECD countries. In the Confucian culture and competitive academic atmosphere, the learning time for students is too long, especially after-school homework time for students.

### 4.2. Nonlinear Effects of Learning Time on Academic Performance

Based on OLS multiple regression models, as shown in Table 3, the research found that among all control variables, male students performed significantly better than female students in math and science. Moreover, female students performed significantly better than male students in reading, indicating that gender plays an important role in different subject learning. The data also show that students in urban areas performed significantly better than those in rural areas, and students in private schools performed significantly better than those in public schools. Students in schools with high-quality teachers performed better than those in schools with normal-quality teachers. Moreover, family background and individual cognitive skills also significantly influence student achievement.

Regarding student learning time, the data of Models 1, 3, and 5 shows that learning time has a significant positive effect on math and science, indicating that increased time commitment promotes student academic achievement and that learning time is a necessary condition for improving subject performance. However, learning time does not have a significant effect on reading performance; it is possible that the benefit of a single increase in learning time is not significant for reading performance. Model 2, Model 4, and Model 6 are models after the inclusion of the quadratic term of learning time, no matter for mathematics, science, or reading. The coefficients of the primary and secondary terms are significant, and the coefficient of the second term is significantly negative, indicating that the effect of learning time on the achievement of each subject may be inverted U-shaped; there should be the best marginal benefit of learning time on academic achievement improvement. Therefore, the specific subsequent threshold regression models should be measured.

### 4.3. Seeking Optimal Learning Time: Threshold Regression Estimation

Based on Hansen’s idea of threshold regression [32], this research examines whether there is a threshold effect on the impact of students’ learning time on academic achievement. The existence of a threshold means that the average learning time of students leads to a jump after reaching the threshold value, which may be an increase or decrease in learning efficiency after reaching a certain learning time, and if there is a double threshold effect, the optimal range of learning time can be obtained. According to Table 4, Table 5 and Table 6, the result shows that for math, science, and reading, there is a threshold effect, and there is indeed a nonlinear effect of learning time on students’ academic performance. The difference in the coefficient of effect before and after the threshold value reveals that there is an optimal learning time for each subject.

First, for mathematics (Table 4), both single and double threshold effects were significant, and the effect of learning duration on mathematics showed high benefit before the threshold and low benefit or even ineffective after the threshold. Additionally, there was a positive effect on students’ mathematics performance at learning duration below 200 min in mathematics subjects (β = 0.879, *p* < 0.001), but with significantly lower facilitation benefit than the period of 200–240 min (β = 1.207, *p* < 0.001), indicating that increasing the time spent on mathematics subjects does have a facilitative effect and that reaching a certain amount of learning time (200 min/week) is very necessary. However, if the learning time becomes longer than 240 min, the effect on students’ mathematics achievement is negative (β = 1.207). However, the coefficient is not significant, which indicates that mathematics learning time above 240 min actually has no effect on mathematics achievement improvement with the reasons of students’ learning fatigue, diminishing marginal benefits, etc. The empirical results suggest that the optimal length of learning time for mathematics is 200–240 min per week.

Regarding the results for science, as shown in Table 5, it can be found that the single-threshold effect was not significant; afterward, the double-threshold effect was further tested, and the result was significant, with thresholds of 440 and 520 min, respectively. The coefficient of the effect of increased learning time in science on students’ academic achievement was significantly positive (*β* = 0.188, *p* < 0.001) when the learning time was less than 440 min, but the coefficient of the effect of learning time on students’ science achievement was significantly positive when crossing the inflection point of 440 min, the coefficient of the effect of learning time on science achievement increased to 0.325 (*p* < 0.001) rapidly. However, after exceeding 520 min, the coefficient of learning time significantly becomes negative (*β* = −0.029, *p* < 0.001), indicating that for science learning, exceeding the 520 min/week learning time on science not only makes nonsense but can also possibly cause a negative result. The optimal length of learning time for science is 440–520 min per week, and science requires more learning time than mathematics to achieve better results.

Furthermore, as for students’ reading performance, according to the results shown in Table 6, the single threshold effect is significant; however, the double threshold effect is not significant. The single threshold value is 200 min, and the coefficient of the effect of reading time on reading achievement is significantly positive (*β* = 0.706, *p* < 0.001) when the learning time is less than 200 min/week, but when crossing the inflection point of 200 min, the learning time has a significantly negative effect on academic achievement (*β* = −0.097, *p* < 0.001), which indicates that the optimal time for reading learning is 200 min/week. In order to improve reading performance, 200 min per week is essential.

Finally, by analyzing the relationship between total learning time and academic performance in each subject in Table 4, Table 5 and Table 6, it can be found that the threshold estimates the value of the total learning time and the impact on each subject are different. For example, for mathematics, the optimal learning time is 1720–1760 min/week. For science, the optimal learning time is less than 1620 min. For reading, the optimal learning time is 1720–1760 min/week. By considering all of the optimal learning time periods of the three subjects, the optimal total learning time for all subjects remains in the range of 1620–1760 min/week, and there will be negative effects if the learning time is longer or shorter than this duration.

### 4.4. Heterogeneity Analysis of the Impact of Learning Time on Students at Different Levels

This research investigates the heterogeneity of the effect of learning time on students’ academic performance through quantile regression, which is used to determine the marginal effect of learning time on academic performance for students at different levels of academic ability, and thus helps to differentiate academic performance improvement strategies for different groups.

**Figure 2:**
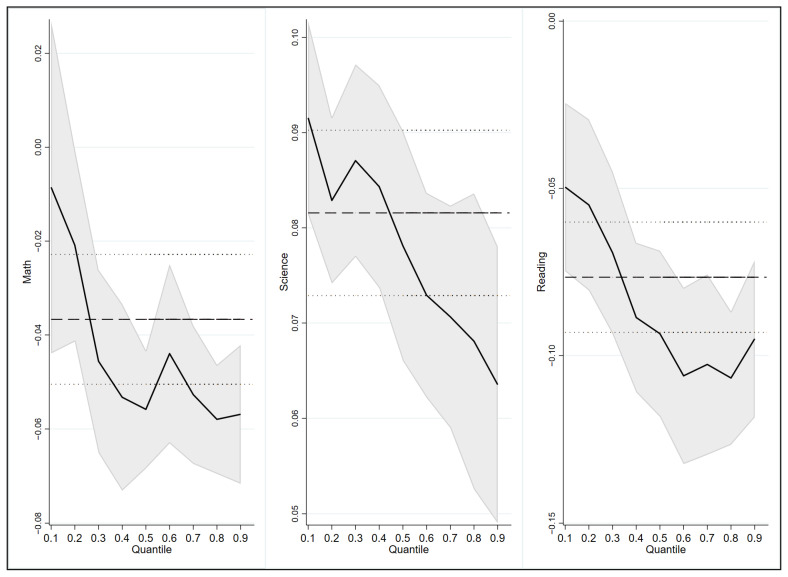
Results of the quantile regression of learning time on students with different levels of academic ability.

As shown in Figure 2, the regression results for the nine quantile points of 0.1–0.9 were obtained by using quantile regression estimation, and the estimated coefficients of the five quantile points were linked to form a trend graph as a way to reflect the differences in the changes in the benefits of learning time for students at different levels (academic ability). The three curves in Figure 2 represent the relationship between time investment in math, science, and reading and academic performance in these three subjects. The curves shifting right represent the coefficient of influence of the learning time investment for each subject on students’ academic achievement with better academic performance.

In the standardized quantile regression model QR, whether for math, science, or reading, the effect of learning time investment on students’ academic achievement at different levels follows the principle of diminishing marginal benefit. Therefore, the increase in learning time investment does not consistently improve students’ academic performance; there is actually variability in the effect on students at different academic levels. Therefore, the overall increase in learning time is more effective for students who are academically disadvantaged; however, it is not particularly effective in improving the performance of students who are already performing very well academically. As shown in Figure 2, for students who are academically disadvantaged (the last 20% to 40% of students), especially for the last 25% of students in math, the increase in learning time has a significant positive effect. With the time commitment increases, the curve shifts to the right and begins to become negative, indicating that it makes sense for the last 25% of students to increase their learning time; however, for the first 75% of students, the effect is not that distinct. Similarly, for the last 40% of students in science and the last 30% of students in reading, it is crucial for them to invest more learning time, and the marginal benefit of learning time on academic achievement is very high. At the same time, it is found that for the students who are in the top 50% of academic achievement, it is not that necessary to increase learning time to strengthen their performance. Furthermore, the benefits of such high-intensity in the form of time “in-roll” for performance improvement are already very weak. Instead, the marginal benefits of learning outcomes should be improved by enhancing the effectiveness of learning.

It cannot be denied that there may be a self-selection effect in the findings of this research because the students who performed not that well are likely to have insufficient cultural capital or less attention from home or school. They may have a relatively low level of commitment to learning or have not reached the optimal threshold of learning time, as studied above. With this background, the increase in learning time guarantees the acquisition of knowledge for the students effectively. Moreover, the students who have good academic performance are those who already have a surplus of academic ability; they have more academic expectations and motivation and may have a relatively sufficient time commitment [33], so they do not need to continue to increase their time commitment. An important conclusion of this research is that for students with unexpected academic performance, it is necessary to supervise the time investment in learning. In contrast, for the students with accepted academic performance, excessive “in-roll” does not bring additional benefits, and even time-consuming learning strategies such as extra-curricular tutoring and repetitive problem-solving are not very effective. It is more important to promote their development by focusing on students’ emotional health, learning strategies, and comprehensive literacy development.

### 4.5. Affect Mechanism of Learning Time on Students’ Academic Performance

A two-step regression was conducted to test the mediating roles of the attitude toward school (learning activities) and subjective well-being (sense of belonging to school), and the results are displayed in Table 7. The primary term coefficient of learning time was significantly positive, and the secondary term coefficient was significantly negative, indicating an inverted U-shaped relationship between the effect of learning time on the attitude toward school (learning activities) and subjective well-being (sense of belonging to school). This means that learning time investment within a certain time frame can effectively increase students’ learning activities and sense of belonging to the school, but excessive time spent on learning will lead to burnout and also affect students’ mental health negatively [34]. Moreover, attitude toward school (learning activities) and subjective well-being (sense of belonging to the school) were significantly and positively related to students’ academic performance. Some research suggested that psychological factors such as student well-being are positively associated with students’ academic performance [35]. This suggests that too many learning objectives affect these two types of non-cognitive states of students, thus hindering their academic performance improvement.

## 5. Conclusions and Recommendations

### 5.1. Conclusions

According to the result of the data analysis, it can be found that there are differences in the length of learning time students have by background, region, and country. Compared with the students who come from the city and public schools, the students from rural and private schools have longer learning times (around 4% longer). There is no association between average school quality and student learning time, suggesting that schools with higher quality teachers do not rely on the style of “in-roll” to create a “quality brand”. In terms of international comparisons, the average length of learning time for students in the four Chinese provinces and cities is about 17% higher than in OECD countries. This is similar to the findings of Yang and Zhao’s study [28], in which many Chinese families choose to increase their children’s learning time investment outside the classroom in order to improve their children’s competitiveness among their peers. Moreover, influenced by Confucianism culture, most Chinese children take working hard as a Chinese virtue; based on this background, the length of learning of Chinese students is generally higher than that of European and American countries [36]. As four provinces and cities in China are relatively developed in education and economic development, compulsory education in these areas is relatively more competitive. Chinese students are overburdened with schoolwork, and the decision-making mechanism of students’ time allocation is very complicated; influenced by the complex social environment, a large amount of out-of-school learning time takes up the time for rest or other comprehensive literacy development that students should have.

Moreover, the effect of learning time on achievement across subjects may be inverted U-shaped; thus, increased learning time does not have a single linear contribution to academic achievement, similar to the results of most studies [37,38]. In addition, according to the data analysis, it was found that there is a learning time duration with optimal marginal benefits on academic achievement. The optimal learning time for learning math, science, and reading is suggested to be 200–240 min per week, 440–520 min per week, and about 200 min per week, respectively. However, the problem of the learning burden has plagued the development of compulsory education in China. Moreover, too much learning load is not only detrimental to the improvement of students’ academic performance but also has negative effects on physical health and psychological health, such as significantly reducing sleep time and exercise time and also increasing the incidence of myopia [39,40]. In addition, overload learning time inevitably causes anxiety and depression among students, which also affects students’ learning efficiency. Excessive time spent on a certain subject affects the performance of other subjects; therefore, the reasonable allocation of learning time for each subject is also crucial.

Thirdly, the impact of learning time investment on the academic performance of students at different levels follows the principle of diminishing marginal benefit, and it is very necessary to increase the learning time for students with inferior academic performance (ranking of last 20−40%). In contrast, for the students whose academic performance is in the top 50%, there is no need to extend the learning time; the study time extension actually has very limited benefit in promoting their achievement. However, Chinese students not only need to obey their schools and parents but also face fierce competition from their peers, resulting in a lack of autonomy in their after-school learning time [28]. It is well recognized that for different students, prolonging learning time is not always conducive to improving academic performance [41]. This research can help educators to further understand that the appropriate optimal learning time should be arranged for students of different statuses to help improve their academic performance scientifically.

Lastly, it is demonstrated that learning time affects student academic performance by influencing students’ attitudes toward school (learning activities) and subjective well-being (sense of belonging to school). Within a certain range of learning time, there is a positive relationship between student engagement in learning and attitudes toward school (learning activities) and subjective well-being (sense of belonging to school), but after a threshold, there is a negative effect. The decline in students’ well-being and subjective learning attitudes affects students’ learning outcomes negatively [42], which in turn affects student academic achievement negatively. It has been shown that excessive learning time after school not only negatively affects students’ academic performance directly but also affects academic performance through non-cognitive factors such as students’ emotions and attitudes. Therefore, the benefits of extending learning time should not be exaggerated, and it is crucial to develop a more scientific learning strategy [13].

### 5.2. Policy Implications

According to the research findings, it should be realized that the students’ learning time should be allocated according to the scientific standard. This research found that the relationship between learning time investment and students’ learning effectiveness is in an inverted U-shape, and there is an optimal range of students’ learning time, which indicates that too much investment in time will not necessarily lead to better outcomes in academic performance, and the results of this research could provide an empirical basis for the “double reduction” policy to a certain extent. However, reducing the learning load or reducing the length of learning does not necessarily lead to poorer learning outcomes for students. The Carroll School Learning Model considers that school learning efficiency, which is the ratio between the actual time spent and the time needed to be spent, can be established by controlling learning time [19]. Therefore, reducing unnecessary learning time investment is necessary to enhance learning efficiency and learning effectiveness. 

Moreover, students at different academic achievement levels should adopt different learning time allocation schemes. The findings showed that learning time allocation has a heterogeneous effect on students at different academic achievement levels, and it is essential to extend learning time for students with low academic achievement. Therefore, adopting the same learning time allocation model for students at different academic levels is a very inefficient way because the top students are very efficient learners who are able to acquire knowledge and skills quickly and therefore do not need to be allocated extra learning time. Their spare time needs to be spent on other comprehensive literacy development [43]. For students who have difficulties in studying, they may not be able to keep up with the progress according to the lesson schedule in the curriculum, and they need to allocate more time for pre-reading and consolidation, so it is necessary and effective to allocate learning time differently for students with different learning abilities.

Lastly, teachers should keep the principle of scientificity, simplification, and pertinence in the design of students’ after-school assignments. Based on the fact that extended learning time does not have a positive impact on student achievement, teachers should try to avoid simple, repetitive, and punitive tasks when assigning after-school homework. Furthermore, too much workload negatively affects subjective well-being as well as attitudes toward learning activities and depletes students’ positive emotions, and these negative non-cognitive aspects further hinder the improvement of students’ academic performance.

### 5.3. Practical Recommendations

For school leaders, the pressure from schools’ enrollment rate should not be a restriction for the leaders to consider the students’ mental health. The excessive homework burden goes against the rules of learning, and it is detrimental to the development of student’s cognitive and non-cognitive abilities. Moreover, schools need to improve the traditional teacher evaluation and assessment system by setting an upper limit on the amount of homework; the headteacher of the class is responsible for coordinating among different subject teachers and should be assured that the final sum of homework for all subjects can meet scientific standards. 

The social stakeholders, especially those professionals who have the responsibility of inspecting, supervising, and guiding the school, education administration agencies, and other education departments, need to strengthen the monitoring of students’ learning time allocation. It is also necessary for them to guide the improvement of school examination methods and contents. The content of primary and secondary school exams in most regions is still based on memorization, and this kind of exam content leads students to devote more time to learning; however, it cannot develop students’ independent and autonomous thinking skills or critical thinking skills, etc.

Moreover, it is crucial for teachers to improve their teaching quality and cultivate differentiated learning engagement strategies for different students. When the teaching quality has been improved, and students’ comprehension becomes better, the necessary learning time for students will be shorter, so it is necessary to improve teaching quality and enhance students’ comprehensive literacy in order to improve the students’ learning efficiency and save time [44].

For students’ parents, it is necessary to be deeply aware that high investment in learning time does not mean good academic performance. Parents should not only focus on their children’s academic performance, and grades should not be the only criterion to evaluate whether their children are good or not. Parents need to pay more attention to developing students’ self-learning skills so that they can complete academic tasks on their own, make better use of their time, and improve their learning efficiency while ensuring necessary sleeping and physical activity time.

## 6. Limitations

There are some limitations to this research. Firstly, since the PISA study is not a tracking study, we may still have endogeneity problems in the model due to the omission of certain variables. Secondly, the PISA study still uses self-reports of students’ learning time, and selectivity bias caused by students’ recall may lead to overestimation or underestimation of the results.

## Figures and Tables

**Table 1:** Description of variables and descriptive statistics.

Variables		Variable Description	Minimum Value	Maximum Value	Average Value	Standard Deviation
Dependent variables	Math scores	Item Response Theory (IRT) Estimates Probability Distributions of Discipline Performance	207.03	863.75	605.88	78.66
	Science scores		216.14	859.59	606.89	81.85
	Reading scores		248.10	847.85	574.95	86.90
Independent variables	Math learning time	Minutes/week	30.00	1350.00	283.57	105.93
	Language learning time		30.00	1600.00	360.40	169.14
	Science learning time		30.00	1250.00	266.67	93.14
	Total learning time		285.00	3000.00	1932.57	394.92
Control variables	Gender	Female = 0, Male = 1	0.00	1.00	0.51	0.50
	Urban and Rural Classification	Rural = 0, Urban = 1	0.00	1.00	0.62	0.49
	School Type	Private school = 0, Public school = 1	0.00	1.00	0.87	0.34
	School Quality	The proportion of teachers with graduate degrees in the school is distinguished as a dichotomous variable	0.00	1.00	0.52	0.50
	Family socioeconomic and cultural status index	According to the three syntheses of parents’ occupational status, education level, and household ownership	−4.68	3.10	−0.25	1.08
	Metacognitive level (comprehension and memory skills)	PISA test item synthesis	−1.64	1.50	0.27	0.97
Mediating variables	Attitude toward school (learning activities)	PISA test item synthesis	−2.537	1.084	0.161	0.926
	Subjective well-being (sense of belonging to the school)	PISA test item synthesis	−3.258	2.756	−0.146	0.908

**Table 2:** The differences in the length of student learning with different backgrounds.

Variables		Gender		Urban and Rural		School Type		School Quality		International Comparison	
		Female	Male	Rural	City	Public	Private	General	Quality	China	OECD
Learning time (minute)	Average value	1930	1934	1983	1901	1920	2016	1971	1897	1932	1650
	Standard deviation	393	405	419	376	392	407	415	371	394	387
	T	−0.513		9.816 ***		7.934 ***		9.186		60.377 ***	
	Conhen’s d	0.026		0.208		0.245		0.189		0.637	

Note: *** *p* < 0.001.

**Table 3:** OLS regression results of the effect of learning time on academic achievement.

Explanatory Variables		Mathematics		Science		Reading	
		Model 1	Model 2	Model 3	Model 4	Model 5	Model 6
Control Variables	Gender	15.133 *** (2.061)	14.917 *** (2.043)	18.437 *** (2.090)	17.984 *** (2.066)	−5.738 *** (2.154)	−5.836 ** (2.133)
	Urban and Rural Classification	4.564 * (2.249)	4.820 * (2.227)	8.057 *** (2.251)	8.667 *** (2.223)	9.070 *** (2.369)	9.299 *** (2.342)
	School Type	−10.560 *** (2.843)	−10.688 *** (2.802)	−6.720 * (2.834)	−5.389 (2.811)	−8.224 ** (3.002)	−7.606 * (3.006)
	School Quality	31.420 *** (2.210)	32.598 *** (2.194)	32.968 *** (2.201)	34.328 *** (2.157)	36.179 *** (2.298)	37.928 *** (2.289)
	Family socioeconomic and cultural status index	17.592 *** (1.057)	17.276 *** (1.046)	16.574 *** (1.100)	15.782 *** (1.085)	21.164 *** (1.131)	20.902 *** (1.118)
	Metacognitive level (comprehension and memory skills)	16.390 *** (1.114)	16.097 *** (1.100)	21.244 *** (1.135)	20.671 *** (1.121)	24.390 *** (1.166)	23.986 *** (1.151)
Independent variable	Learning time (subject)	0.037 *** (0.010)	0.219 *** (0.038)	0.089 *** (0.006)	0.264 *** (0.021)	0.004 (0.013)	0.272 *** (0.048)
	Learning time (subject) squared items		−0.0002 *** (0.000)		−0.0002 *** (0.000)		−0.0004 *** (0.000)
intercept distance		585.51 *** (4.844)	554.23 *** (7.454)	554.49 *** (4.223)	520.31 *** (5.650)	564.58 *** (5.534)	523.04 *** (8.963)
Fit	*R* ^2^	0.195	0.202	0.256	0.273	0.267	0.275
	F	182.37	166.02	262.12	251.15	267.24	242.64

Note: * *p* < 0.05, ** *p* < 0.01, *** *p* < 0.001.

**Table 4:** Threshold regression estimation results for the length of learning time in mathematics.

Threshold Variables		Single Threshold		Double Threshold		
		Q_i_ ≤ φ	Q_i_ > φ	Q_i_ ≤ φ_1_	φ_1_ < Q_i_ ≤ φ_2_	Q_i_ > φ_2_
Mathematics	Threshold of subject learning time	0.879 *** (0.448)	−0.042 *** (0.008)	0.879 (0.044)	1.207 (0.190)	−0.039 (0.010)
	Intercept distance	423.123 *** (8.720)	594.648 *** (3.825)	423.853 *** (8.701)	304.491 *** (44.634)	593.4337 *** (4.518)
	Threshold value φ	200 min/week		200–240 min/week		
	Total Learning Hours Threshold	0.117 *** (0.006)	−0.010 *** (0.0028)	0.1130 *** (0.006)	6.4890 *** (1.031)	−0.0100 *** (0.002)
	Intercept distance	388.475 *** (9.833)	605.639 *** (6.825)	393.4060 *** (10.463)	568.3450 *** (42.546)	606.3460 *** (6.813)
	Threshold value φ	1760 min/week		1720–1760 min/week		
Other variable control		YES		YES		

Note: *** *p* < 0.001.

**Table 5:** Threshold regression estimation results for hours of study in science subjects.

Threshold Variables		Single Threshold		Double Threshold		
		Q_i_ ≤ φ	Q_i_ > φ	Q_i_ ≤ φ_1_	φ_1_ < Q_i_ ≤ φ_2_	Q_i_ > φ_2_
Science	Threshold of subject learning time	0.188 (0.008)	−0.013 (0.010)	0.188 *** (0.008)	0.325 *** (0.080)	−0.029 * (0.013)
	Intercept distance	517.119 (3.572)	598.590 (6.296)	517.417 *** (3.569)	437.569 *** (38.178)	609.434 *** (9.465)
	Threshold value φ	440 min/week		440–520 min/week		
	Total Learning Hours Threshold	0.109 *** (0.007)	−0.012 *** (0.002)	0.087 *** (0.010)	−0.167 (0.139)	−0.012 *** (0.002)
	Intercept distance	394.909 *** (11.814)	608.647 *** (6.206)	420.454 *** (14.380)	840.106 *** (222.229)	608.440 *** (6.203)
	Threshold value φ	1620 min/week		<1530 min		
Other variable control		YES		YES		

Note: * *p* < 0.05, *** *p* < 0.001.

**Table 6:** Threshold regression estimation results for the length of study in reading subjects.

Threshold Variables		Single Threshold		Double Threshold		
		Q_i_ ≤ φ	Q_i_ > φ	Q_i_ ≤ φ_1_	φ_1_ < Q_i_ ≤ φ_2_	Q_i_ > φ_2_
Read	Threshold of subject learning time	0.706 *** (0.042)	−0.097 *** (0.010)	0.706 *** (0.042)	−0.059 (0.037)	−0.043 * (0.018)
	Intercept distance	433.028 *** (8.183)	584.849 *** (4.338)	433.003 *** (8.178)	576.537 *** (10.141)	559.498 (8.031)
	Threshold value φ	200 min/week		<200 min		
	Total learning Hours threshold	0.120 *** (0.006)	−0.011 (0.003)	0.117 *** (0.006)	5.994 *** (1.084)	−0.011 *** (0.003)
	Intercept distance	433.028 *** (8.183)	582.081 *** (7.169)	362.617 *** (10.996)	375.698 *** (19.654)	582.728 *** (7.160)
	Threshold value φ	1760 min/week		1720–1760 min/week		
Other variable control		YES		YES		

Note: * *p* < 0.05, *** *p* < 0.001.

**Table 7:** Results of the analysis of the mechanism of study time on academic performance.

Explanatory Variables		Intermediate Variables		Math Scores	
		Attitude Toward School (Learning Activities)	Subjective Well-Being (Sense of Belonging to the School)		
Independent variable	Learning time	0.406 *** (0.081)	0.305 *** (0.080)		133.821 *** (6.491)
	Squared items of learning time	−0.054 *** (0.012)	−0.050 *** (.012)		−17.990 *** (0.991)
Intermediate variables	Attitude towards school: learning activities			7.532 *** (0.774)	5.918 *** (0.783)
	Subjective well-being: a sense of belonging to the school			1.772 * (0.789)	1.141 (0.795)
Control variables		YES	YES	YES	YES
Fit	*R* ^2^	0.019	0.020	0.181	0.227
	F	34.769	36.088	434.373	353.240

Note: * *p* < 0.05, *** *p* < 0.001.

## Data Availability

Data will be made available upon request.
